# Myosin Storage Myopathy in *C*. *elegans* and Human Cultured Muscle Cells

**DOI:** 10.1371/journal.pone.0170613

**Published:** 2017-01-26

**Authors:** Martin Dahl-Halvarsson, Malgorzata Pokrzywa, Manish Rauthan, Marc Pilon, Homa Tajsharghi

**Affiliations:** 1 Department of Pathology, University of Gothenburg, Sahlgrenska University Hospital, Gothenburg, Sweden; 2 Department of Chemistry and Molecular Biology, University of Gothenburg, Gothenburg, Sweden; 3 School of Health and Education, Div Biomedicine and Public Health, University of Skovde, Skovde, Sweden; University of Cincinnati, UNITED STATES

## Abstract

Myosin storage myopathy is a protein aggregate myopathy associated with the characteristic subsarcolemmal accumulation of myosin heavy chain in muscle fibers. Despite similar histological findings, the clinical severity and age of onset are highly variable, ranging from no weakness to severe impairment of ambulation, and usually childhood-onset to onset later in life. Mutations located in the distal end of the tail of slow/ß-cardiac myosin heavy chain are associated with myosin storage myopathy. Four missense mutations (L1793P, R1845W, E1883K and H1901L), two of which have been reported in several unrelated families, are located within or closed to the assembly competence domain. This location is critical for the proper assembly of sarcomeric myosin rod filaments. To assess the mechanisms leading to protein aggregation in myosin storage myopathy and to evaluate the impact of these mutations on myosin assembly and muscle function, we expressed mutated myosin proteins in cultured human muscle cells and in the nematode *Caenorhabditis elegans*. While L1793P mutant myosin protein efficiently incorporated into the sarcomeric thick filaments, R1845W and H1901L mutants were prone to formation of myosin aggregates without assembly into striated sarcomeric thick filaments in cultured muscle cells. In *C*. *elegans*, mutant alleles of the myosin heavy chain gene *unc-54* corresponding to R1845W, E1883K and H1901L, were as effective as the wild-type myosin gene in rescuing the null mutant worms, indicating that they retain functionality. Taken together, our results suggest that the basis for the pathogenic effect of the R1845W and H1901L mutations are primarily structural rather than functional. Further analyses are needed to identify the primary trigger for the histological changes seen in muscle biopsies of patients with L1793P and E1883K mutations.

## Introduction

The class II conventional muscle myosin exists as a hexameric protein composed of two myosin heavy chain (MyHC) subunits and two pairs of non-identical light chain subunits [1, 2]. MyHC is a molecular motor that converts chemical energy into mechanical force, and is indispensable for body movement and heart contractility. MyHCs associate into dimers through a coiled-coil interaction along the long tail domains. Dimerization of two MyHCs results in a polar structure with two distinct regions, which provide the motor and filament-forming functions (S1A Fig). The amino terminus forms a globular head domain that binds to actin and ATP, which is required for motor activity [3]. The elongated α-helical coiled-coil C-terminal rod domain exhibits filament-forming properties that assemble into thick filaments of the sarcomeres located in the A-band [1] (S1A Fig). MyHC is the major constituent of the sarcomeric thick filaments, and therefore has an essential structural function. Myosin binding protein C (MyBPC), located in the central region of the A-band, promotes the polymerisation of the sarcomeric thick filaments.

There are several striated muscle MyHC isoforms encoded by different genes and expressed in a tissue and developmental specific manner [4–8]. Slow/β-cardiac MyHC, encoded by *MYH7*, is expressed in slow, type 1 muscle fibres as well as in the ventricles of the heart.

Mutations in *MYH7*, predominantly located in the globular myosin head, are an important cause of hypertrophic and dilated cardiomyopathy. Mutations in the rod light meromyosin (LMM) region have been associated with skeletal myopathies with several distinct morphological and clinical phenotypes, depending on the mutated residue at the tail region [9]. Mutations located in the distal end of the tail of slow/ß-cardiac MyHC corresponding to exons 37–40 of *MYH7* have been associated with myosin storage myopathy (MSM), a protein aggregate myopathy associated with the characteristic subsarcolemmal accumulation of material consisting mainly of MyHC in muscle fibres [9, 10]. The first mutation identified in MSM was a dominant missense mutation changing the highly conserved and positively charged arginine at position 1845 to the uncharged, aromatic tryptophan (R1845W) [9, 10]. This mutation is found in several unrelated cases with MSM, indicating a critical role of residue arginine 1845 for slow/β-cardiac MyHC [10–14]. The recurrent L1793P mutation has been reported in patients with MSM with or without cardiomyopathy [15, 16] and the dominant missense mutation H1901L is the likely cause of MSM in a Saudi Arabian kindred with several affected individuals [17]. The first autosomal recessive MSM associated with a homozygous missense mutation (E1883K) was reported in three siblings who developed hypertrophic cardiomyopathy with cardiac failure that was lethal in two cases [18].

Mutations in *MYH7* located in exon 32–36 in the mid region of the MyHC rod are associated with Laing distal myopathy [9, 19–22]. Unlike myosin storage disease, the muscle pathological changes in most reported cases with Laing distal myopathy are variable and unspecific [22–25]. Protein aggregates are usually not seen in muscle biopsy form these patients [9].

The assembly of MyHC filaments involves both the proper folding of α-helices into coiled-coils, and the assembly of these coiled-coils into filaments. Coiled-coils are two-stranded protein motifs, where each strand is an *α*-helix with a repeating heptad of residues (*a-b-c-d-e-f-g*). Positions *b*, *c*, *e*, *f*, *g* are exposed on the surface of the protein, where the side chains are available to interact with other proteins. Defects in any steps in formation of MyHC filaments, caused by mutations, may result in improper filament formation leading to pathological conditions. Mutations in the rod region of myosin can affect the ability of the protein to form stable and functional thick filaments, based on the amino acid change, the position in the heptad repeat-motif and the location in the rod region. Missense mutations associated with MSM (L1793P, R1845W, E1883K and H1901L) are located in the outer position (*b*, *c*, *f*), where the side chains are available to interact with other myosin dimers (S1B Fig). Moreover, these mutated residues are located within or close to the 29-residue assembly competence domain (ACD), in the C-terminus coiled-coil rod region of MyHC, which is known to be critical for the proper assembly of sarcomeric myosin rod filaments [26]. Biochemical and biophysical characterization of the effects of L1793P, R1845W and H1901L have suggested adverse effects of the mutations in the ability of the protein to form stable and functional thick filaments [27].

Here, we investigated the pathogenesis of L1793P, R1845W and H1901L MSM mutations and their impact on myosin assembly and sarcomere formation in transfected human skeletal muscle cells in culture. In addition, the effects of the R1845W, E1883K and H1901L mutations on muscle function were evaluated in the nematode *Caenorhabditis elegans*.

## Results

### Morphological analysis in transfected human tissue culture

**Fig 1:**
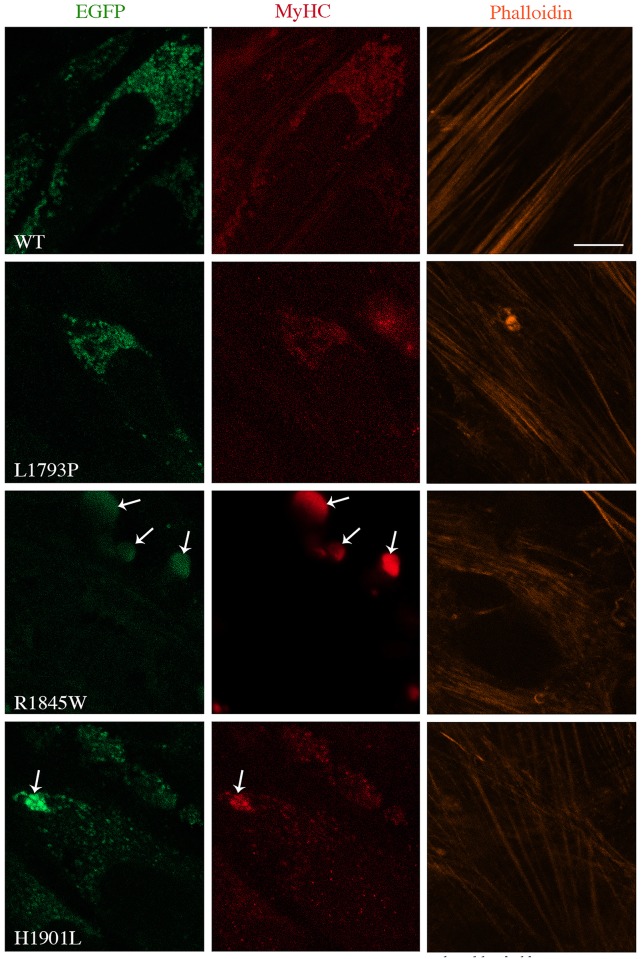
Morphological analysis of WT and slow/ß-cardiac MyHC mutants in human myoblasts in culture. Myoblasts transfected with WT and mutant EGFP-tagged slow/ß-cardiac MyHC constructs were imaged 15 h after transfection. Immunostaining was performed for MyHC (red) and F-actin (orange). Similar pattern of punctate myosin structure was found in cells transfected with WT and L1793P mutant. Arrows indicates the dense MyHC accumulation at the peripheral area of transfected myoblasts with E1845W and H1901L mutants. MyHC in cells transfected with H1901L appeared in punctate-like structures and aggregates. The bars represent 10 μm.

We examined the behaviour of selected slow/β-cardiac MyHC mutations associated with MSM in a physiologically relevant context by transfecting the human muscle cells in culture. To characterise the behaviour of wild-type (WT) or mutant MyHC in a differentiated myotube system, transfected human myoblasts were differentiated for 1 and 3 days and analysed for filament formation and cytoplasmic distribution by confocal microscopy. Untransfected differentiated myotubes stained with MyHC antibody were used as control (S1C Fig). In addition, fluorescent phalloidin was used to visualize filamentous actin and examine the integration of the transfected myosin into endogenous actin filament in myoblasts. While WT and L1793P mutant myosin proteins formed similar punctate myosin structures distributed through the cytoplasm of transfected myoblasts, formation of dense bodies appeared to be the dominant phenotype of the R1845W mutant protein (Fig 1). The R1845W mutant protein failed to form the nascent myosin structures, as seen with WT and L1793P myosin. Although the mutant H1901L protein formed punctate myosin structures distributed through the cytoplasm, appearance of the aggregates localised at the peripheral area of transfected myoblasts were frequent. The punctate myosin structures and aggregate bodies seen in the R1845W and H1901L transfected myoblasts were stained with MyHC antibody, indicating that they were composed of MyHC (Fig 1).

**Fig 2:**
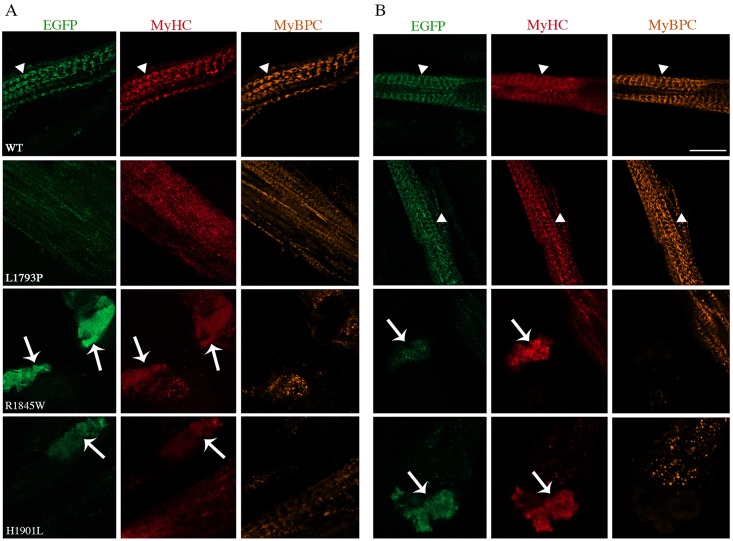
Co-immunofluorescence analysis of 1- and 3-day differentiated myotubes. Double immunostaining was performed for MyHC (red) and MyBPC (orange) for 1-day (A) and 3-day (B) differentiated myotubes. The repetitive well-structured sarcomere can be seen clearly in 1- and 3-day differentiated myotubes transfected with WT and in 3-day differentiated myotubes with L1793P mutant EGFP-tagged slow/ß-cardiac MyHC constructs (arrowheads). Cells transfected with WT and L1793P mutant were co-immunostained for MyHC and MyBPC. Arrows indicate the accumulation of myosin in 1- and 3-day differentiated myotubes transfected with E1845W and H1901L mutants. Note the myosin accumulations are devoid of MyBPC staining. The bars represent 10 μm.

**Fig 3:**
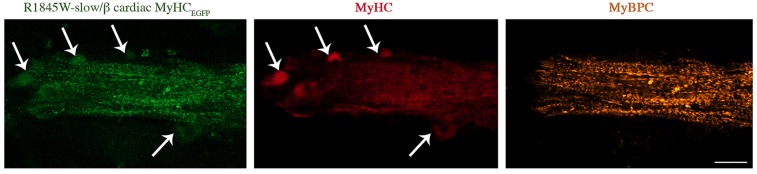
Co-immunofluorescence analysis of 3-day differentiated myotubes. Double staining was performed for MyHC (red) and MyBPC (orange) for 3-day differentiated myotubes transfected with E1845W mutant EGFP-tagged slow/ß-cardiac MyHC construct. Arrows indicate the myosin accumulations, which appear as eccentric areas of myobtubes devoid of MyBPC activity. Note the appearance of nascent thick filaments and absence of well-structured sarcomere. The bars represent 10 μm.

We investigated the proper localization of transfected myosin protein at the A-bands and the alternating band pattern of sarcomere in differentiated myotubes. Overall, the staining patterns for MyHC and MyBPC (another component of sarcomeric thick filaments) were similar between WT and L1793P slow/β-cardiac MyHC mutant transfected cells (Fig 2A and 2B). 1-day differentiated myotubes transfected with WT myosin showed the well-structured alternating band pattern of sarcomere. However, appearance of nascent thick filaments was more frequent in 1-day differentiated myotubes transfected with L1793P slow/ß-cardiac MyHC mutant. 3 days after differentiation, L1793P slow/ß-cardiac MyHC mutant protein behaved like the WT protein. It was efficiently incorporated into the sarcomeric thick filaments of elongated myotubes and demonstrated the formation of expected alternating band pattern of the sarcomeric A-bands (Fig 2A and 2B). This suggests that both WT and L1793P slow/ß-cardiac MyHC mutant proteins are correctly localised to the sarcomeric A-band. Double-immunostaining for MyHC and MyBPC demonstrated a regular appearance of the myofibrils in the cells transfected with WT and L1793P slow/ß-cardiac MyHC mutant proteins (Fig 2A and 2B). In contrast, cells transfected with R1845W and H1901L mutant myosin proteins appeared cytopathic. Formation of the distinctive alternating band patterns of sarcomere and A-bands in three-day differentiated myotubes transfected with R1845W and H1901L mutant myosin was absent (Figs 2B and 3). However, the appearance of nascent thick filaments was observed in the myotubes transfected with R1845W and H1901L mutant myosin compared to WT or L1793P mutant (Fig 2), indicating delayed myofibrilogenesis. Large aggregates stained with MyHC were observed in 1 and 3 days differentiated myotubes transfected with R1845W and H1901L myosin mutants. The myosin aggregates appeared as eccentric areas located at the peripheral regions of the myotubes and they were not incorporated into nascent thick filaments (Figs 2 and 3). Double-immunostaining for MyHC and MyBPC showed that myosin aggregates in cultured muscle cells transfected with mutant R1845W and H1901L myosin proteins were devoid of MyBPC staining (Figs 2 and 3), indicating that they were not incorporated into thick filament component. In addition, infrequent filamentous R1845W mutant MyHC, incorporated into the thick filament of the nascent non-striated sarcomeres was observed in three-day differentiated myotubes (Fig 3).

### Motility assay in transgenic worm

**Fig 4:**
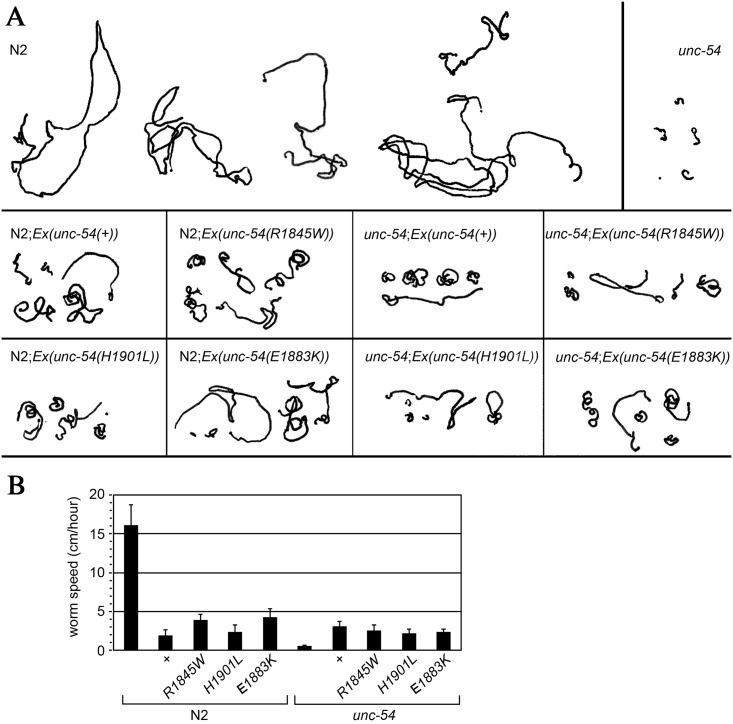
Analysis of *C*. *elegans* motility assay. (A) 1-hour motility traces of five different worms of each genotype. (B) Histogram illustrating the mean worm speed during 1 hour at room temperature. Each bar represents the mean of five worms. Error bars correspond to standard error of the mean.

Normal worms (N2) showed a high degree of spontaneous motility, moving approximately 17 cm per hour. In contrast, *unc-54* mutant worms, which lack a functional copy of the major *C*. *elegans* myosin heavy chain expressed in body wall muscles [28] were paralyzed, moving 0.5 cm/hour (Fig 4). The N2 worms with transgenic expression of wild-type MyHC B (N2; *Ex unc-54(*±*)*) showed a reduction in motility, a phenomenon that we previously observed [28], and which is likely caused by excess expression from the multi-copy extrachromosomal arrays that carry the transgene [29]. The same effect was obtained when using *unc-54* transgene with either the R1854W, E1892K or H1910L mutations (N2; *Ex [unc-54(R1854W)]*), (N2; *Ex [unc-54(E1892K)]*), (N2; *Ex [unc-54(H1901L)]*), respectively (Fig 4).

As expected, and in agreement with our previous work [28], introduction of the wild-type UNC-54 in the *unc-54* null mutant partially rescued the motility defect. Importantly, the three mutant alleles, R1854W, E1892K and H1910L were as effective as the wild-type UNC-54 in rescuing the mutant (Fig 4). This is a strong evidence that they retain at least some degree of functionality.

## Discussion

Disruption in the proper folding of newly synthesized or pre-existing proteins as well as in pathways responsible for refolding or degradation of misfolded proteins may lead to protein aggregation. Such protein aggregates are the characteristic pathomorphological feature in a wide variety of acquired and hereditary diseases including skeletal myopathies.

Mutations in the middle and distal part of the slow/β-cardiac MyHC rod LMM region have been associated with several distinct morphological and clinical phenotypes depending on the location of the mutated residue. Myosin storage myopathy and Laing distal myopathy are the two most important skeletal muscle manifestations of *MYH7* mutations located in the coiled-coil rod region. However, it is unclear why MSM, a protein aggregate myopathy, associated with the characteristic subsarcolemmal accumulation of MyHC in muscle fibres [9, 10], and Laing distal myopathy are associated with different muscle pathologies. Both L1793P and R1845W mutations have been reported in several unrelated cases, indicating that the highly conserved L1793 and R1845 are hotspot amino acids [10–14] [15, 16]. In addition, the H1901L mutation has been reported to cause MSM in a family with several affected individuals [17]. Muscle biopsy of MSM patients with R1845W and H1901L mutations has revealed the pathognomonic changes of myosin storage in the muscle fibres of affected individuals [10–14, 17]. The L1793P mutation has been reported in two unrelated families with intra- and interfamilial striking phenotypic variability [15, 16]. In one family, this mutation has been associated with MSM presenting as neonatal hypotony [16]. In another family a woman showed the features of MSM and cardiomyopathy [15]. However, her daughter presented with cardiac failure already at age 3 months without any skeletal muscle involvement [15].

Given that R1845W and H1901L mutations are located at the outer *f* position, it can be predicted that these mutations alter the ability of myosin to properly assemble into thick filaments. However, the L1793P mutation is located at the *c* position that may stabilize the structure of the protein. Several *in vitro* studies have been performed to investigate molecular mechanisms involved in the pathogenesis of diseases caused by mutations in the rod region of muscle MyHC [27, 30, 31]. The analyses of assembly and stability of filament formation of the L1793P, R1845W and H1901L proteins have indicated that these mutations cause structural, thermodynamic and functional differences [27]. While the L1793P mutation decreased the ability of the protein to properly assemble, the R1845W and H1901L mutations affected the myosin protein in the same manner with formation of atypically large and unusually stable filaments [27].

In accordance with a previous study [27], our present results suggest that there are various pathobiological mechanisms associated with these mutations, which ultimately cause the same disease phenotype. By transgenic expression of mutant L1793P, R1845W and H1901L myosin proteins in human muscle cells in culture, dominant adverse effects could be demonstrated including formation of myosin aggregates. While R1845W and H1901L mutations were prone to formation of myosin aggregates, the L1793 was not associated with overt structural changes. This is in line with the phenotypes observed in the previous study demonstrating the formation of large and stable filaments associated with the R1845W and H1901L mutations but not the L1793P [27]. R1845W and H1901L mutations are located close to the 29-residue ACD, in the C-terminus rod region of MyHC, which is known to be critical for the proper assembly of sarcomeric myosin rod filaments. The myosin aggregates in transfected cells with R1845W or H1901L mutations were located at the periphery of the myotubes, and were not predominantly incorporated into nascent thick filaments. This is consistent with the morphological phenotype of characteristic subsarcolemmal accumulation of material seen in the muscle fibres of affected individuals with MSM [9, 10]. Furthermore, the stored bodies did not assemble with MyBPC, the other component of sarcomeric thick filament. These observations suggest that aberrant accumulation of MyHC is due to disturbed interactions between mutated MyHC and thick filaments. Thus, R1845W and H1901L mutations may cause improper filament formation through disturbed incorporation into the sarcomere, resulting in aberrant accumulation of unassembled proteins. In contrast, the L1793P mutation that is situated at distance from the ACD might cause other primary effects on myosin and muscle function leading to phenotypic variability. Failure to detect L1793P myosin accumulations may suggest that our cell assays have timing limitations that may only reveal histological and structural changes associated with the initial stages of the disease. The basis of the primary effects of L1793P mutation on histological feature remains open and requires further studies.

**Fig 5:**
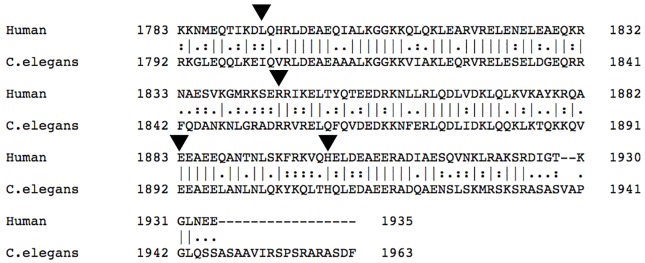
Alignment of a part of the distal end of the tail of slow/ß-cardiac MyHC (human) and UNC-54 (*C*. *elegans*) using Pairwise Sequence Alignment, EMBOSS Needle. Arrowheads indicate the residues L1793, E1845, E1883 and H1901 of slow/ß-cardiac MyHC in human, corresponding to I1802, E1854, E1892 and H1901 of UNC-54 in *C*. *elegans*.

We generated transgenic *C*. *elegans* to simulate MSM and evaluated the functional effects of the E1883K, R1845W and H19001L mutations. Unlike L1793P, R1845W and H19001L mutations with dominant mode of inheritance, the E1883K mutation is associated with an autosomal recessive MSM in a family with three siblings who developed hypertrophic cardiomyopathy with cardiac failure that was lethal in two cases [18]. Thus, assessment of the functional effects of the E1883K mutation was of interest. However, as the L1793 is not conserved in *C*. *elegans* MyHC B (*unc-54*), the transgenic worm of this variant was not generated (Fig 5).

The experiments with *C*. *elegans* must be interpreted with caution given the fact that the wild-type *unc-54* transgene, provided as part of a multicopy extrachromosomal array, is detrimental to wild-type worms and only partially rescues the *unc-54* mutant. Nevertheless, it is clear from the present study that the three studied mutations, namely R1845W, 1883K and H1901L, retain significant function since they appear to be just as efficient as the wild-type version of the protein in rescuing the *unc-54* null mutant worms. The results from the experiments with *C*. *elegans* as well as cell transfection assays may indicate that the pathogenic effect of the R1845W and H1901L mutations are structural rather than functional. Furthermore, the results from motility assay in worm rule out a primary functional consequence of E1883K mutation.

The use of animal models in combination with *in vitro* studies allows investigation of the disease mechanisms and clarifies the functional and structural properties of slow/β MyHC mutations. Although the results from the present and previous studies further our understanding of MSM and the pathogenesis leading to protein aggregation, more subtle genetic interventions are needed to get insight into the mechanisms governing the formation of protein aggregations and the cardiac and/or skeletal muscle involvement. In conclusion, our findings suggest that changes in the structural properties of slow/β-cardiac MyHC caused by E1845W and H1901L mutations may be regarded as the primary trigger of the disease and basis for the histological changes seen in muscle biopsies of patients with MSM. Further studies will be required to elucidate the pathogenic basis of the L1793P and E1883K mutations, which did not give any detectable structural or functional phenotypes in our study.

## Materials and Methods

### DNA constructs

The full-length wild-type slow/β-cardiac MyHC cDNA fragment (transcript GI: 85567607, protein accession: P12883), cloned into a pCR-XL-TOPO vector, was obtained through (imaGenes Gmbh, Berlin, Germany). The cloned cDNA fragment was cut with *XhoI* and *EcoRI* restriction enzymes and gel purified using a QIAquick Gel extraction kit (Qiagen, Hilden, Germany). The gel-purified slow/ß-cardiac MyHC fragment with XhoI (5’) and EcoRI (3’) restriction sites was sub-cloned into the *XhoI* and *EcoRI* restriction sites of the pEGFP-N1 to generate WT-slow/ß-cardiac MyHC_EGFP_. All slow/ß-cardiac MyHC mutant constructs were generated in the pEGFP-N1 backbone (Clontech, USA). EGFP-tagged slow/ß-cardiac MyHC mutants (R1845W, L1793P and H1901L) were generated through site-directed mutagenesis and cloning into *E*. *coli* using a Quick- ChangewSite-Directed Mutagenesis kit (Stratagene, UK), as previously described [32]. WT- and mutant-slow/ß-cardiac MyHC_EGFP_ constructs were sequenced to verify the complete slow/ß-cardiac MyHC coding sequence and correct introduction of the desired mutations.

The R1854W, E1892K and H1910L mutations of myosin heavy chain B (corresponding to R1845W, E1883K and H1901L in human) were generated by modifying the pPD5.41 plasmid, which carries the complete coding sequence and regulatory regions of the *C*. *elegans* myosin heavy chain B (*unc-54*) gene (GI:156399) sub-cloned as a 9 kb *Hpa*I fragment into the *Sma*I site of pBS-KS(+) (Stratagene, La Jolla, CA), as previously described [28]. Briefly, oligonucleotides were used with the Stratagene QuikChange Site-Directed Mutagenesis Kit, according to the manufacturer’s instructions and as previously described [28], to replace the R1845, E1892 and H1901 with the W1845, R1892 and L1901 codons, respectively. DNA sequencing of these constructs confirmed presence of the desired mutations. The WT and mutated constructs used for microinjection experiments thus included the entire coding sequence as well as the promoter region of the myosin heavy chain B gene and only differed at WT/mutated position, respectively.

### Culture of human myoblasts and transfection

The standardised human myoblast batches were provided by MYOSIX through a collaborative programme with Association Francaise contre les Myopathies (AFM). The skeletal muscle cells from a donor with no clinical signs of muscle disease were enzymatically isolated and cultured, as previously described [33, 34]. All reagents were purchased from Invitrogen Life Technologies (Invitrogen, Carlsbad, CA, USA), unless otherwise specified. The human myoblasts in their second passage were cultured and differentiated at 37°C in a humidified 5% CO2 atmosphere, as previously described [32].

100×103 cells of human myoblasts were plated on chamber slides in medium without antibiotics one day prior to transfection. Cells were transfected at 85%–90% confluence using Lipofectamine 2000^™^ transfection reagent, according to the manufacturer’s instructions and as previously described [32]. To induce the differentiation of transfected myoblasts, cells were washed in differentiated medium, composed of DMEM supplemented with 5% horse serum, 10 mg/ml insulin and 10 mg/ ml penicillin, and further incubated in this medium for one to three days (D1 and D3 differentiated myotubes). The transfection experiments were conducted in duplicate and repeated at least five times for each transfected slow/ß-cardiac MyHC construct.

### Immunofluorescence analysis and confocal microscopy of transfected myoblasts and differentiated mytotubes

At given time points, cells plated on chamber slides were washed three times in phosphate-buffered saline (PBS) and fixed with 4% formaldehyde for 10 min. Free aldehyde groups were blocked with 50mM NH4Cl for 10 min and cells were permeabilised in PBS containing 0.1% Triton X-100 for 4 min. Cells were incubated with anti-MyHCs (developmental studies hybridoma bank, Iowa, USA) and anti-MyBPC (BB88) for 60 min in the dark followed by 60 min incubation with secondary antibodies. Secondary antibodies goat anti rabbit IgG conjugated to DyLight^®^ 549 and anti goat anti mouse IgG conjugated to DyLight^®^ 649 were purchased from Vector Laboratories Inc, Burlingame, CA, USA. Filament actin was visualised using Alexa Fluor^®^ 555-conjugated phalloidin obtained from Molecular Probes, NY, USA. Stained cells were mounted in Prolong^®^ Gold antifade reagent with DAPI to highlight cell nuclei and imaged using a Confocal LSM800 microscope from Zeiss, using a 63× objective with Immersol 518F oil. Images were processed using Photoshop software (Adobe, USA).

### Nematode strains and culturing

General methods for the culture, manipulation, and genetics of *C*. *elegans* were as described [29]. All strains were cultured at 20°C. Strains used in this study were the wild-type Bristol N2 and CB190 which bears the *unc-54 (e190)* null mutation. Both strains were obtained from the *C*. *elegans* Genetics Centre (Minneapolis, MN).

### Generation of transgenic worms

Transgenic worms of the E1845W = E1854W, E1883K = E1892K and H1901L = H1910L mutations associated with either autosomal dominant or recessive MSM were generated. As the L1793 is not conserved in *C*. *elegans* myosin heavy chain B (*unc-54*), transgenic worms of this variant was not generated. Ten different genotypes were investigated: (1) N2: the wild- type reference strain; (2) *unc-54 (e190)*: a *unc-54-*null mutant, previously isolated in a screen for uncoordinated mutants [35, 36]; (3) N2; *Ex*(*unc-54*(±)): wild type with the *unc-54* gene on an extrachromosomal array; (4) N2; *Ex* (*unc- 54*(R1854W)): wild type with the mutated *unc-54(R1854W)* gene on an extrachromosomal array; (5) N2; *Ex* (*unc- 54*(E1892K)): wild type with the mutated *unc-54(E1892K)* gene on an extrachromosomal array; (6) N2; *Ex* (*unc- 54*(H1910L)): wild type with the mutated *unc-54(H1910L)* gene on an extrachromosomal array; (7) *unc-54; Ex* (*unc- 54*[±]): *unc-54* null mutant with the wild-type *unc-54* gene on an extrachromosomal array; (8) *unc-54; Ex* (*unc- 54*(R1854W)): *unc-54* null mutant with the mutated *unc-54*(*R1854W*) gene on an extrachromosomal array; (9) *unc-54; Ex* (*unc- 54*(E1892K)): *unc-54* null mutant with the mutated *unc-54*(E1892K) gene on an extrachromosomal array; (10) *unc-54; Ex* (*unc- 54*(H1910L)): *unc-54* null mutant with the mutated *unc-54*(H1910L) gene on an extrachromosomal array. Note that genotypes 8, 9 and 10 represent the closest genetic equivalent to animals homozygous for *R1854W*, *E1892K* and *H1910L*.

Transformation of *C*. *elegans* was performed using methods described previously [37] [28]. Transgenic lines carrying the microinjected DNA on extrachromosomal arrays were established from GFP-positive F2 progeny of injected worms and maintained by picking GFP-positive animals over subsequent generations, as previously described [28]. At least two lines of each transgenic genotype were generated with similar results.

### Motility assay

Motility assay was performed as previously described [28]. Briefly, individual 1-day-old adult worms were transferred to 9 cm nematode growth medium plates and allowed to move and graze freely on the *E*.*coli* lawn at room temperature. The animal positions were recorded every second minute to generate traces over 1 hour for each worm. At least five animals of each strain were analysed.

## Web Addresses

The sequence information was obtained from the National Centre for Biotechnology Information (http://www.ncbi.nlm.nih.gov/); the protein alignment was constructed by use of Pairwise Sequence Alignment, EMBOSS Needle (http://www.ebi.ac.uk/Tools/psa/emboss_needle/). BLAST analyses were performed using the NCBI BLAST Web site (http://www.ncbi.nlm.nih.gov/blast/bl2seq/wblast2.cgi). GenBank accession numbers for genomic *unc-54* sequence was J01050 (GI: 156399) and for cDNA *unc-54* sequence was NM_061195 (GI: 17509400).

## Supporting Information

S1 Fig Positions of the MSM mutations in the slow/β-cardiac MyHC rod domain. The globular head domain is shown in red. (A) The neck domain is shown in yellow and the C-terminal LMM segment of the rod domain is shown in orange. The heptad repeat motif forms the structural basis for coiled-coil dimer of the β-MyHC. (B) The positions of slow/β-cardiac MyHC mutations associated with MSM in the heptad repeat motif are indicated. (C) Differentiated human myotubes stained with MyHC show clear A-bands (white bracket).
(TIF)
