# Supplementary figures and images for: Myosin Storage Myopathy in C. elegans and Human Cultured Muscle Cells
Source: PLoS One. 2017 Jan 26;12(1):e0170613. doi: 10.1371/journal.pone.0170613 (PMC5268365; doi:10.1371/journal.pone.0170613)

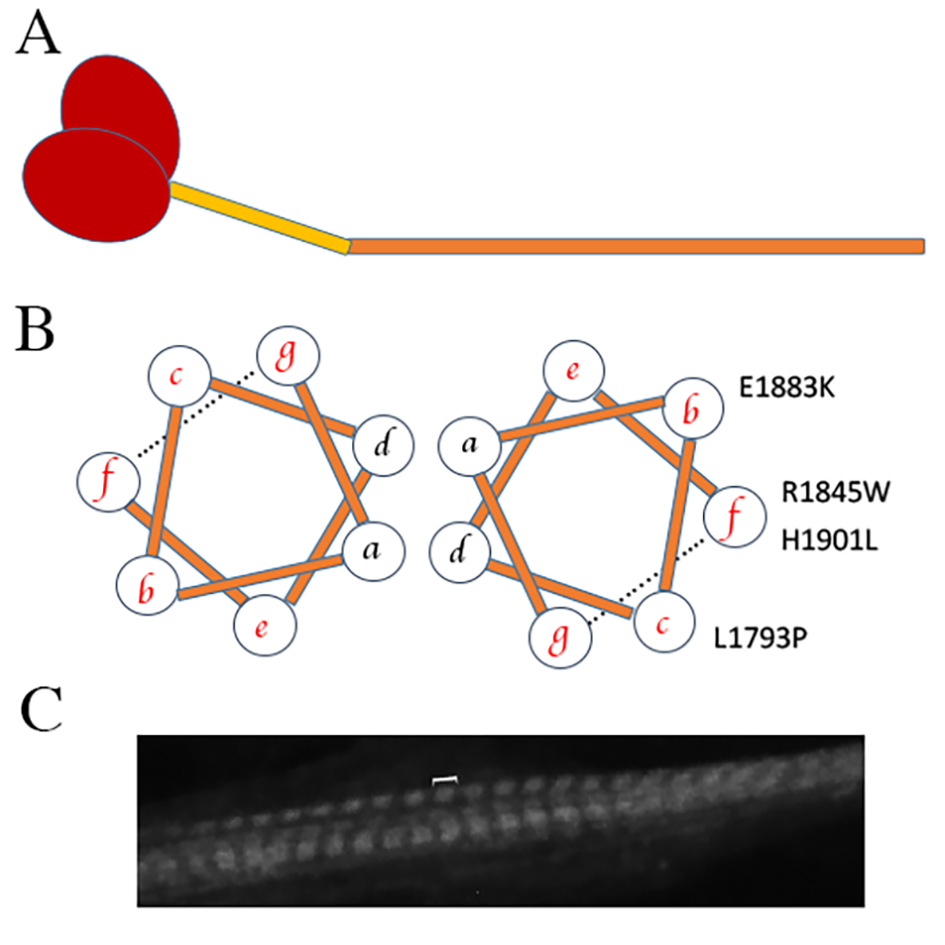

Supplement: S1 Fig — The globular head domain is shown in red. (A) The neck domain is shown in yellow and the C-terminal LMM segment of the rod domain is shown in orange. The heptad repeat motif forms the structural basis for coiled-coil dimer of the β-MyHC. (B) The positions of slow/β-cardiac MyHC mutations associated with MSM in the heptad repeat motif are indicated. (C) Differentiated human myotubes stained with MyHC show clear A-bands (white bracket). (TIF) [file pone.0170613.s001.tif]
